# The exotic behavior of the wave evolution in Lévy crystals within a fractional medium

**DOI:** 10.1038/s41598-023-48110-8

**Published:** 2023-11-25

**Authors:** Z. Zakeri, M. Solaimani, L. Lavaei, S. A. A. Terohid

**Affiliations:** 1 Department of Physics, Hamedan Branch, Islamic Azad University, Hamedan, 1574365181 Iran; 2 Department of Physics, Qom University of Technology, Qom, 3718146645 Iran

**Keywords:** Nonlinear optics, Solitons

## Abstract

We investigate a traveling Gaussian wave packet transport through a rectangular quantum barrier of lévy crystals in fractional quantum mechanics formalism. We study both standard and fractional Schrödinger equations in linear and nonlinear regimes by using a split-step finite difference (SSFD) method. We evaluate the reflection, trapping, and transmission coefficients of the wave packet and the wave packet spreading by using time-dependent inverse participation ratio (IPR) and second moment. By simultaneously adjusting the fractional and nonlinear terms, we create sharp pulses, which is an essential issue in optoelectronic devices. We illustrate that the effects of barrier height and width on the transmission coefficient are strangely different for the standard and fractional Schrödinger equations. We observe fortunately soliton-like localized wave packets in the fractional regime. Thus, we can effectively control the behavior of the wave evolution by adjusting the available parameters, which can excite new ideas in optics.

## Introduction

Pulsed laser technology has made it possible to experimentally study the wave packet formation and dynamics in different systems^1^. In both atomic and molecular systems, spatially localized wave packet has been observed^2^. The localization degree of the wave function can be tuned using different factors such as disorder^3^, magnetic field^4^, nonlinearity^5^, etc. Also, the localization of light waves^6^ and ultracold atoms^7^ have so far been investigated. Wave transport and evolution are essential parts of the different fields of physics, such as wave packet dynamics in grapheme^8^, tunneling through nanostructures^9^, photon-atom interactions^10^, soliton dynamics^11^, multiphoton ionization^12^, MOS devices^13^, etc.

Since Laskin^14^ introduced the fractional dimension idea and studied the fractional Schrödinger equation for a few cases, such as quarkonium and one-dimensional fractional oscillator^15^, this issue has attracted the researchers’ attention to apply the fractional dimension idea in the various fields of sciences. Due to potential applications, the fractional dimension idea in differential equations, especially in the Schrödinger equation, has been successfully used to interpret different phenomena and systems. Solitons^16–18^, Rabi oscillations^19^, quantum dots^20^, beam propagation dynamics^21^, donor and acceptor properties in semiconductor superlattices^22^, non-crystalline solids^23^, polarons in a quantum well^24^, pseudo-relativistic boson stars^25^, charge transport in large-scale organic polymers such as DNA^26^, optical Bloch oscillation and Zener tunneling^27^, etc. are a few instances of using the fractional dimension idea in physics.

The fractional Schrödinger equations usually arise in the systems where particles interact with each other over long distances^28,29^. By using an aspherical optical cavity, an optical realization of the fractional Schrödinger equation has also been advanced by Longhi et al.^30^. Then, the eigenmodes of a mass-less harmonic oscillator have been obtained^30,31^. In the condensed-matter physics context^32^, Stickler et al. have introduced the one-dimensional Lévy crystals as a candidate for experimentally accessible realization of space-fractional quantum mechanics.

In this paper, the interplay between fractionality and nonlinearity in the Schrödinger equation is investigated because of the novelty and importance of this issue. In fact, the interplay between disorder and nonlinearity has been studied previously^33^, but beam evolution in the nonlinear fractional Schrödinger equation is still not well understood^34^. We use a split-step finite difference method to solve a one-dimensional time-dependent space fractional Schrödinger equation. We precisely describe the technique and present the algorithm. First, we study the reflected and transferred parts of an initial Gaussian traveling wave packet as a function of position and then the reflection, trapping, and transmission coefficient of the one when it is scattered by a rectangular quantum barrier, by varying the magnitude of barrier height, barrier width, fractional and nonlinear parameters, the coefficient of the fractional Laplacian, etc. Finally, we investigate the spreading of the wave packet as a function of time by using the time-dependent inverse participation ratio (IPR) and second moment. Since the fractional Schrödinger equation consists of many novelties that exclusively belong to this equation, nor the standard Schrödinger equation, so we believe that our paper will give new ideas to the researchers of the fractional Schrödinger equation.

## Formalism

We start with the following one-dimensional nonlinear fractional Schrödinger equation

1 $$ i\hbar \frac{\partial }{\partial t}\psi (x,t) = \left[ { - \kappa \frac{{\partial^{\alpha } }}{{\partial \left| x \right|^{\alpha } }} + \gamma \left| {\psi (x,t)} \right|^{2} + V(x)} \right]\psi (x,t), $$

where

2 $$ \int\limits_{ - \infty }^{\infty } {|\psi (x,t)|^{2} dx} = 1, $$

and the Riesz fractional operator for $1 < \alpha \le 2$ is defined as^35^

3 $$ \frac{{\partial^{\alpha } }}{{\partial \left| x \right|^{\alpha } }}\psi (x,t) = \frac{1}{2\cos (\alpha \pi /2)\Gamma (2 - \alpha )}\frac{{d^{2} }}{{dx^{2} }}\int\limits_{ - \infty }^{\infty } {\left| {x - \xi } \right|^{1 - \alpha } \psi (\xi ,t)d\xi } , $$

where $\Gamma$ is the gamma function. The parameter $\gamma$ is the nonlinear coefficient, and the term $\gamma \left| {\psi (x,t)} \right|^{2}$ represents the electron–electron interaction, i.e. the contribution of Colombian repulsion^36^. The coefficient $\kappa$, which can be written as $\kappa = \frac{{\hbar^{2} }}{{2m^{*} }}$ in the standard Schrödinger equation, can model the effect of the effective mass of the electron, so larger $\kappa$ is equivalent to the system with smaller effective mass m^*^. Finally, the term $V(x)$ is the external potential that we define it as a rectangular quantum hetero-structure

4 $$ V(x) = \left\{ \begin{gathered} 0,\,\,\,\,\left| x \right| > L/2 \hfill \\ V_{0} ,\,\,\,\,\left| x \right| \le L/2 \hfill \\ \end{gathered} \right., $$

where $L$ is the width of the quantum barrier. To study a traveling Gaussian wave packet in the fractional medium, we use the following initial function (initial condition) at $t = 0$

5 $$ \psi (x,t = 0) \equiv \exp \left[ { - \frac{{(x - x_{0} )^{2} }}{{2\beta^{2} }} + ikx} \right], $$

where $x_{0}$, $\beta$ and $k$ are the center, the width, and the wave vector of the wave packet, respectively. In some figures of Section "Results and discussions", we assign numerical values to velocity instead of $k$ because $p = \hbar k$ and also $p = m^{*} v$, so $k$ is proportional to velocity.

Now, we use a SSFD (split step finite difference) method to solve Eq. (1) and evaluate the wave packet at later times; also, the reflection, trapping, and transmission coefficients are computed. The SSFD method is a well-developed method^37^ and has the advantages that it is easy to use and unconditionally stable. Its numerical accuracy is also tested for nonlinear Schrödinger equations of 1d, 2d, and 3d^38^.

### Fractional derivative

Let^39^

6 $$ g_{k} = \frac{{\left( { - 1} \right)^{k} \Gamma (\alpha + 1)}}{{\Gamma (\frac{\alpha }{2} - k + 1)\Gamma (\frac{\alpha }{2} + k + 1)}}, $$

where $k = 0, \mp 1, \mp 2,...$ and $\alpha > 1$, so

7 $$ g_{0} \ge 0, $$

and

8 $$ g_{ - k} = g_{k} \le 0,\,\,\,\left| k \right| \ge 1. $$

Now

9 $$ \Delta_{h}^{\alpha } f(x) = \sum\limits_{k = - \infty }^{\infty } {\frac{{( - 1)^{k} \Gamma (\alpha + 1)}}{{\Gamma \left( {\frac{\alpha }{2} - k + 1} \right)\Gamma \left( {\frac{\alpha }{2} + k + 1} \right)}}f(x - kh)} , $$

is the fractional centered difference where $h$ is space step, and then we have

10 $$ - h^{ - \alpha } \Delta_{h}^{\alpha } f(x) = \frac{{\partial^{\alpha } }}{{\partial \left| x \right|^{\alpha } }}f(x) + O(h^{2} ). $$

When $h \to 0$,$\frac{{\partial^{\alpha } }}{{\partial \left| x \right|^{\alpha } }}f(x)$ is the fractional derivative for $1 < \alpha \le 2$.

### Split step method

First, we divide Schrödinger Eq. (1) into two linear

11 $$ i\hbar \frac{\partial \psi (x,t)}{{\partial t}} = - \kappa \frac{{\partial^{\alpha } }}{{\partial \left| x \right|^{\alpha } }}\psi (x,t), $$

and nonlinear parts

12 $$ i\hbar \frac{\partial \psi (x,t)}{{\partial t}} = V(x)\psi (x,t) + \gamma \left| {\psi (x,t)} \right|^{2} \psi (x,t). $$

We solve the linear part by using a Crank-Nicolson schema and solve the nonlinear part exactly. We discretize the position axis (M points) with coordinates $x_{j} = jh$ in which $j = 0,1, \ldots ,M - 1$ and also for time $t_{n} = n\tau$ where $n = 0,1, \ldots ,N - 1$. The position and time steps are $h = \frac{L}{M - 1}$ and τ, respectively. Let $\psi_{j}^{n} = \psi (x_{j} ,t_{n} )$ where $\psi_{{}}^{n}$ has the components $\psi_{j}^{n}$. Then, we solve the linear part by the Crank-Nicolson method. First, we write

13 $$ i\hbar \frac{\partial \psi (x,t)}{{\partial t}} = - \kappa \frac{{\partial^{\alpha } }}{{\partial \left| x \right|^{\alpha } }}\psi (x,t), $$

and then, by using the discretization of space and time, we have

14 $$ i\hbar \frac{{\psi_{j}^{ * * } - \psi_{j}^{ * } }}{\tau } = \frac{1}{2}\kappa h^{ - \alpha } \sum\limits_{k = j - M + 1}^{j} {g_{k} \psi_{j - k}^{ * * } } + \frac{1}{2}\kappa h^{ - \alpha } \sum\limits_{k = j - M + 1}^{j} {g_{k} \psi_{j - k}^{ * } } , $$

where $j = 0,1, \ldots ,M - 1$. Equation (14) can be written as

15 $$ (I - A)\psi^{**} = (I + A)\psi^{*} , $$

where A is the M × M symmetric matrix as

16 $$ A = - \frac{i\tau \kappa }{{2h^{\alpha } }}\left[ \begin{gathered} g_{0} \,\,\,\,\,\,\,\,\,\,\,g_{ - 1} \,\,\,\,\,\,g_{ - 2} \,\,\,\, \cdots \,\,\,g_{ - M + 1} \hfill \\ g_{1} \,\,\,\,\,\,\,\,\,\,\,\,g_{0} \,\,\,\,\,\,\,g_{ - 1} \,\,\,\, \cdots \,\,\,g_{ - M + 2} \hfill \\ \vdots \,\,\,\,\,\,\,\,\,\,\,\,\,\,\,\, \vdots \,\,\,\,\,\,\,\,\,\,\, \vdots \,\,\,\,\,\,\, \ddots \,\,\,\,\,\,\,\, \vdots \hfill \\ g_{M - 1} \,\,\,g_{M - 2} \,\,\,g_{M - 3} \,\,\,\, \cdots \,\,\,\,\,g_{0} \hfill \\ \end{gathered} \right]. $$

Using (15), we have

17 $$ \psi^{**} = (I - A)^{ - 1} (I + A)\psi^{*} , $$

and then

18 $$ \psi^{**} = (2(I - A)^{ - 1} - I)\psi^{*} , $$

so

19 $$ \psi^{**} = (Q^{ - 1} - I)\psi^{*} . $$

We define $\chi = Q^{ - 1} \psi^{*}$, so $Q^{{}} \chi = \psi^{*}$ is an equation set where $\psi_{{}}^{ * }$ is known, $\chi$ is unknown, and $Q^{ - 1}$ is the inverse of $Q = \frac{1}{2}(I - A)$.

### Algorithm

(A) We find $\psi_{j}^{ * }$ for $n = 0,1,2, \ldots$ and $j = 0,1, \ldots ,M$

20 $$ \psi_{j}^{*} = e^{{ - i\left( {V_{j} + \gamma \left| {\psi_{j}^{n} } \right|^{2} \tau } \right)/2}} \psi_{j}^{n} . $$

(B) For $$j = 1,2,...,M - 1$$

21 $$ \psi^{**} = (I - A)^{ - 1} (I + A)\psi^{*} , $$

22 $$ \psi_{0}^{ * * } = \psi_{M}^{ * * } = 0. $$

(C) We put the result of Eq. (21) in the following equation

23 $$ \psi_{j}^{n + 1} = e^{{ - i\left( {V_{j} + \gamma \left| {\psi_{j}^{ * * } } \right|^{2} \tau } \right)/2}} \psi_{j}^{ * * } . $$

Now, Eq. (1) has numerically been solved. Finally, we introduce three physical observables: the reflection (R), trapping (L), and transmission (T) coefficients of the wave packet. We calculate them at some time levels after the initial wave packet collides with the barrier, through

24-1 $$ R = \int\limits_{ - \infty }^{ - L/2} {dx} \left| {\psi (x,t)} \right|^{2} , $$

24-2 $$ L = \int\limits_{ - L/2}^{L/2} {dx\left| {\psi (x,t)} \right|^{2} } , $$

and

24-3 $$ T = \int\limits_{L/2}^{\infty } {dx\left| {\psi (x,t)} \right|^{2} } , $$

where R + L + T = 1._._

Now, to study the spreading of the traveling Gaussian wave packet as a function of time in the Lévy crystal, we calculate the inverse participation ratio (IPR) as^40^

25 $$ IPR(t) = \int\limits_{ - \infty }^{\infty } {dx\left| {\psi (x,t)} \right|^{4} } , $$

and the second moment ($\sigma$) as^40^

26 $$ \sigma^{2} (t) = \int\limits_{ - \infty }^{\infty } {dx\,x^{2} \left| {\psi (x,t)} \right|^{2} } - \left( {\int\limits_{ - \infty }^{\infty } {dx\,x\left| {\psi (x,t)} \right|^{2} } } \right)^{2} . $$

The IPR shows the spatial extent of the electronic wave packet, and estimates the degree of its localization. In fact, the IPR of localized states is larger than the one of delocalized states. The second moment (mean-square displacement) measures the average norm spread around the wave packet center or the degree of the spreading of it. Since $\sigma^{2}$ has the units of the square distance, the localization value in one dimension can be defined as proportional to the second moment^41^. Thus, the second moment of localized states is smaller than that of delocalized states.

## Results and discussions

Here, we study the transfer of a Gaussian traveling wave packet from an external rectangular potential barrier through computing transmission, reflection, and trapping coefficients. For this purpose, we solve Eq. (1) by applying the split step finite difference method on fractional differential equations, which we described in the previous section.

**Figure 1:**
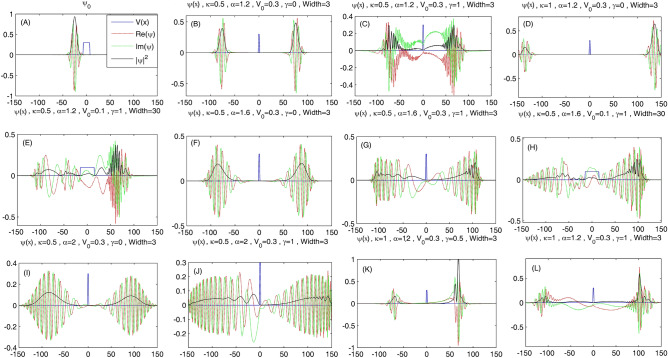
The variation of the Gaussian wave packet as a function of the position x. All panels of this figure consist of the real part, the imaginary part, and the square of the absolute value of the wave packet, and also a typical potential barrier. Panel **A** illustrates the initial Gaussian wave packet, Panels **B** to **J** show the shapes of the reflected and transferred parts of the initial Gaussian wave packet (RP-IGW and TP-IGW) at time 200, and Panels **K** and **L** show these quantities at time 100 (see videos 1 to 11). Panels **B** to **L** are related to the different values of the parameter $\kappa$, the nonlinear parameter $\gamma$, and the fractional parameter $\alpha$ in Eq. (1), as well as the potential height V_0_ and potential width, which have been specified on the corresponding panels.

In Fig. 1, we show the variation of a Gaussian wave packet as a function of the position x. All panels of this figure consist of the real part, the imaginary part, and the square of the absolute value of the wave packet, and also a typical potential barrier. Panel A illustrates the initial Gaussian wave packet, Panels B to J show the shapes of the reflected and transferred parts of the initial Gaussian wave packet (RP-IGW and TP-IGW) at time 200; and Panels K and L show these quantities at time 100, which are different from Panels B to J because at time 200 RP-IGW and TP-IGW collide with the left and right walls of the space region and bounce back. Panels B to L are related to the different values of the parameter $\kappa$, the nonlinear parameter $\gamma$, and the fractional parameter $\alpha$ in Eq. (1), as well as the potential height V_0_ and potential width, which have been specified on the corresponding panels. To see the time evolution of the Gaussian wave packet, we provide some animations. These animations show the wave packet evolution in some limited cases of study in which we properly specify the simulation conditions. We can compare the panels of Fig. 1 with each other, which consist of fractional Schrödinger equation ($\alpha \ne 2$) and non-fractional Schrödinger equation ($\alpha = 2$, which is standard Schrödinger equation) in both cases linear ($\gamma = 0$) and nonlinear ($\gamma \ne 0$) equations. As we can see in Panels B, D, F, and I, related to $\gamma = 0$, the shapes of RP-IGW and TP-IGW, are two Gaussian wave packets like the initial one. Since these panels consist of both cases $\alpha = 2$ and $\alpha \ne 2$, so we conclude that fractionality does not change the Gaussian shapes of RP-IGW, and TP-IGW. From Panels B and F, it is seen that for smaller values of $\alpha$, related to more fractional Schrödinger equation, RP-IGW and TP-IGW more slowly spread, so they are similar to localized states. Comparing Panel I with Panel J, or B with C, we can see that RP-IGW and TP-IGW faster spread for $\gamma \ne 0$ and their shapes are not Gaussian. Panels B and D show that RP-IGW and TP-IGW more rapidly travel in systems with larger values of $\kappa$, related to the smaller values of effective mass m^*^. Finally, Panels K and L, which are at time 100, also show that we can create sharp shock pulses by simultaneously using the fractional and nonlinear terms in the Schrödinger equation. However, as these two panels show, the sharpness of the created shock wave does not increase by further increase of the nonlinear term.

**Figure 2:**
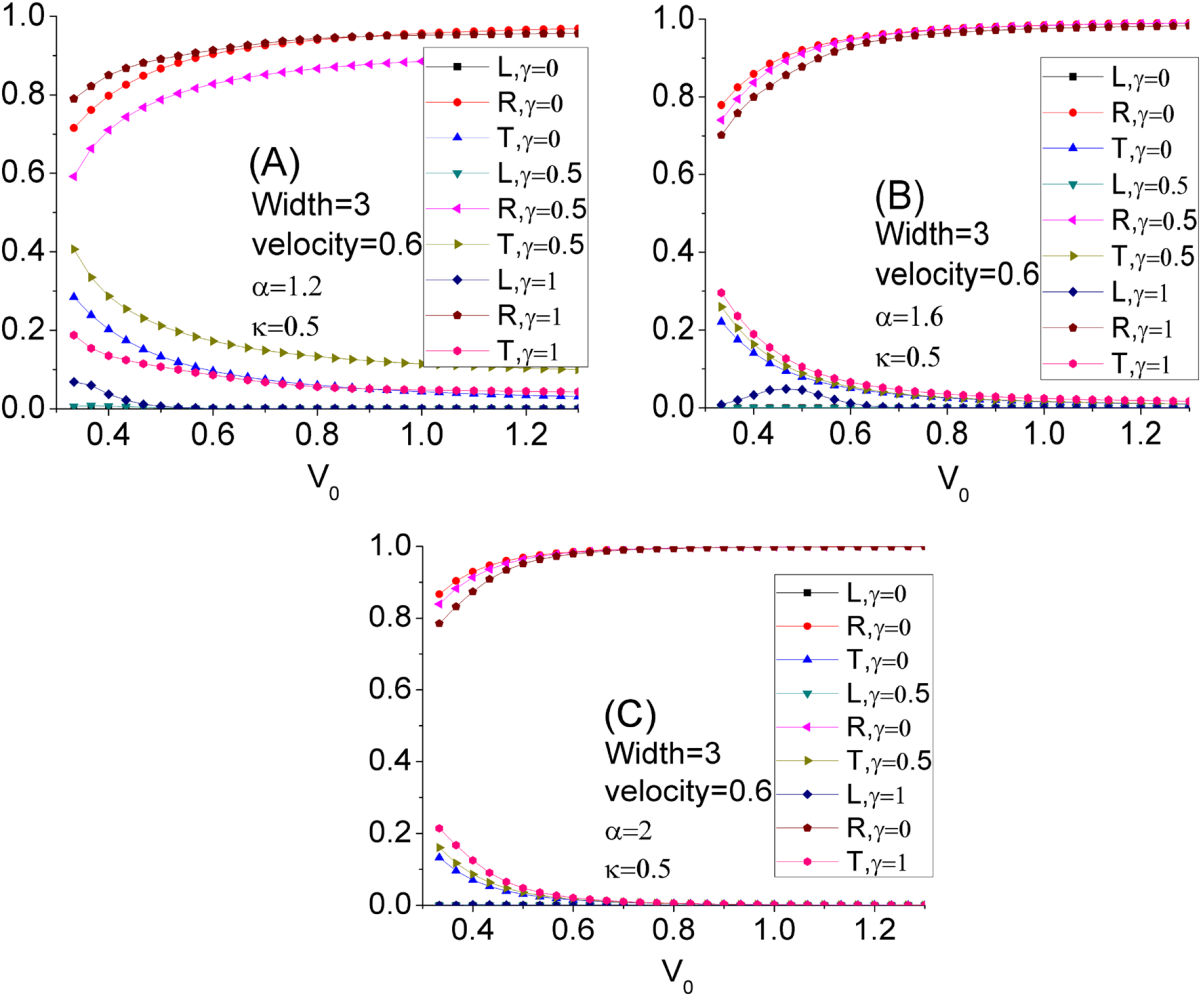
The variation of the reflection (R), the trapping (L), and the transmission (T) coefficients of the Gaussian wave packet as a function of the barrier height V_0_. Panels **A**, **B**, and **C** are related to the fractional parameter values $\alpha$ = 1.2, 1.6, and 2, respectively. In these panels, the effect of the nonlinear parameter $\gamma$(= 0, 0.5, 1) has also been shown.

Figure 2 illustrates the variation of the reflection (R), the trapping (L), and the transmission (T) coefficients of the Gaussian wave packet as a function of barrier height V_0._ Panels A, B, and C are related to the fractional parameter values $\alpha$ = 1.2, 1.6, and 2, respectively. In these panels, the effect of the nonlinear parameter $\gamma$ (= 0, 0.5, 1) has also been presented. In all of these panels, when the value of the barrier height increases, the transmission coefficient decreases and the reflection coefficient increases, which is entirely conspicuous. From these panels, it is seen that the trapping coefficient is non-zero only for the fractional Schrödinger equation (i.e. Panels A and B) when the nonlinear parameter $\gamma$ is large enough (i.e. $\gamma$ = 1). Meantime, by decreasing $\alpha$, we have non-zero L for less barrier height V_0_, so we need less V_0_ to trap some part of the incident wave packet in the barrier for more fractional Schrödinger equation. As Panel A shows, when the nonlinear parameter $\gamma$ increases from 0 to 1, the transmission coefficient first increases and then decreases, but the reflection coefficient first decreases and then increases because the summation of L, R, and T has to be 1. From Panel C, we see that for V_0_ more than about 0.7 (and any $\gamma$), the transmission coefficient becomes zero, at the same time this happens for V_0_ more than about 1.1 in Panel B, and the scenario is entirely different in Panel A because T is non-zero in Panel A for all V_0_ and $\gamma$, especially for $\gamma$ = 0.5. Thus, we can always have the transmission of the wave packet from the barrier with any height for the nonlinear and enough fractional Schrödinger equation. In Panel A, the transmission coefficient T is lowest for $\gamma$ = 1 comparison with $\gamma$ = 0 and $\gamma$ = 0.5 for smaller V_0_, and it is lowest for $\gamma$ = 0 comparison with $\gamma$ = 1 and $\gamma$ = 0.5 for larger V_0_. Thus, to obtain the desired T for a given V_0_, we should choose whether to use the linear or nonlinear fractional Schrödinger equation for more fractional systems.

**Figure 3:**
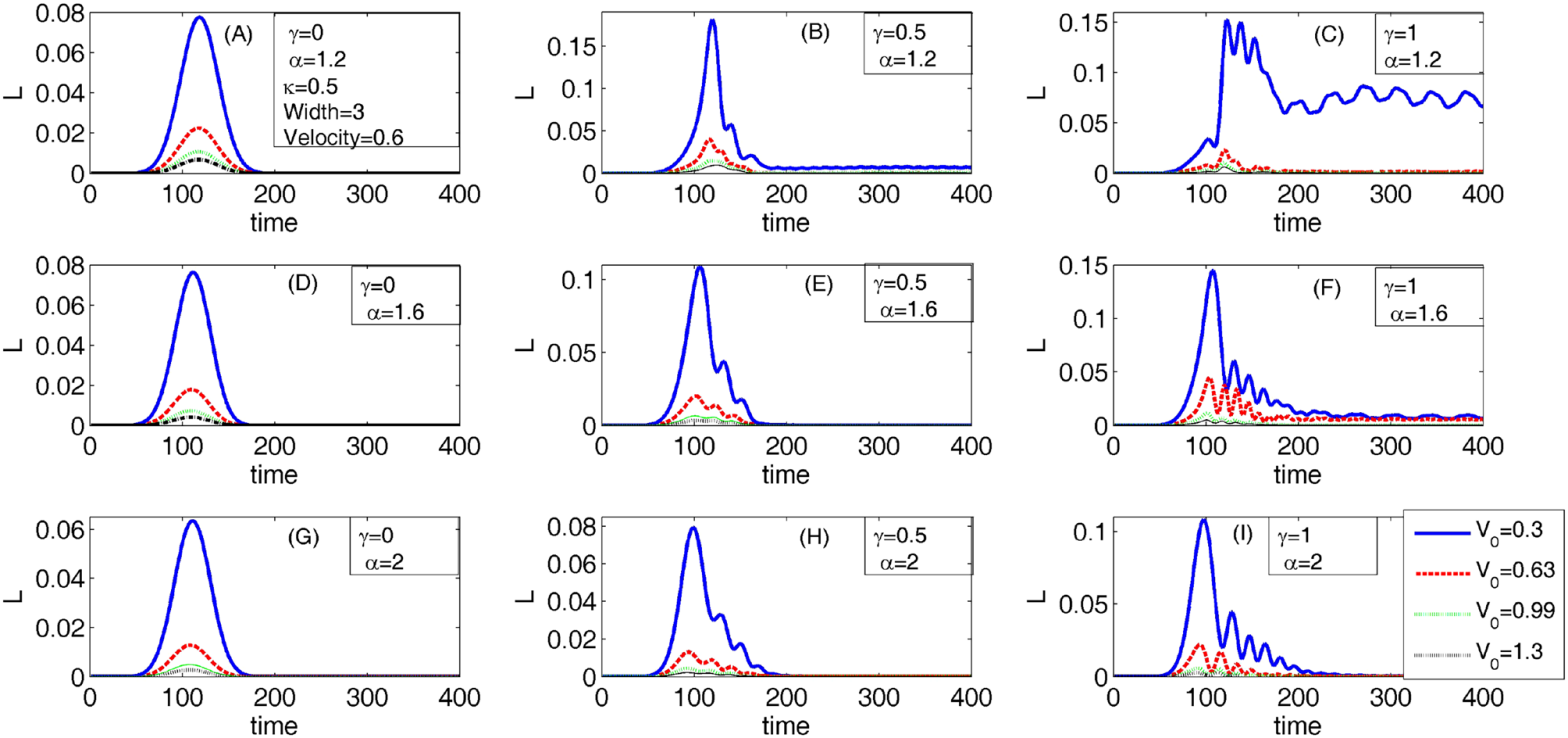
The variation of the trapping coefficient L as a function of the time. Panels **A** to **I** are due to different values of the nonlinear parameter $\gamma$ and fractional parameter $\alpha$, which have been specified in the corresponding panel.

Figure 3 shows the variation of the trapping coefficient L as a function of time. Panels A to I are related to different values of the nonlinear parameter $\gamma$ and fractional parameter $\alpha$, which have been specified on the corresponding panel. As this figure shows, for a given nonlinear parameter $\gamma$, the trapping coefficient decreases by increasing the fractional parameter $\alpha$. However, for a given fractional parameter $\alpha$, the trapping coefficient increases by increasing the nonlinear parameter $\gamma$. On the other hand, the trapping coefficient decreases by increasing the barrier height V_0_. As Panel C shows, the trapping coefficient L has an approximately constant non-zero value after the time t = 200 for V_0_ = 0.3. Therefore, by using a small value of the barrier height (V_0_ = 0.3), the fractional parameter ($\alpha$ = 1.2), and an approximately large value of the nonlinear parameter ($\gamma$ = 1), we can trap some part of the incident Gaussian wave packet in the barrier region, like one happened in Panel A of Fig. 2.

**Figure 4:**
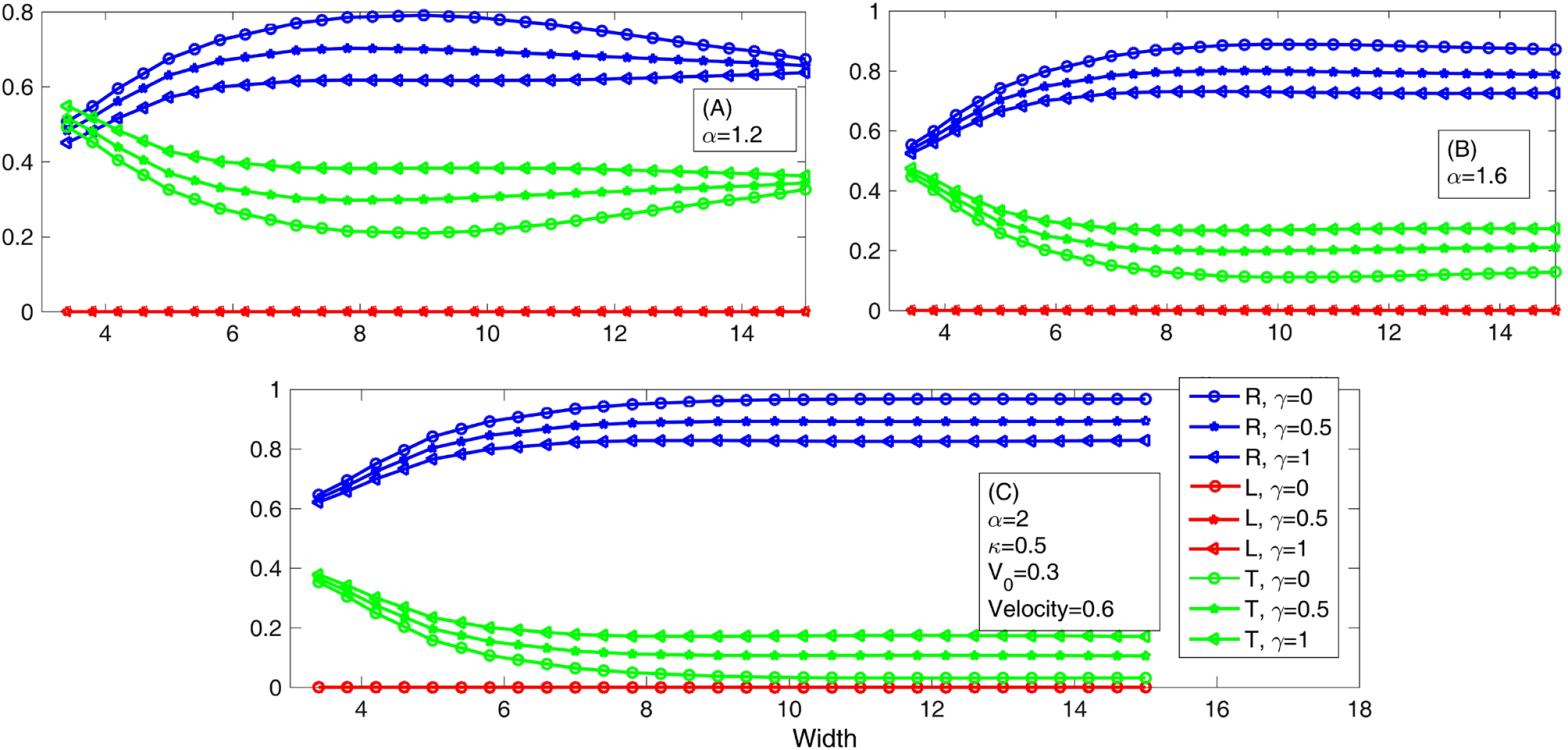
The variation of the reflection, trapping, and transmission coefficients as a function of the barrier width. Panels **A**, **B**, and **C** are related to the fractional parameter values $\alpha$ = 1.2, 1.6, and 2, respectively.

To see the effect of barrier width, we plot Fig. 4, which shows the variation of the reflection, the trapping, and the transmission coefficients as a function of the barrier width. Panels A, B, and C are related to the fractional parameter values $\alpha$ = 1.2, 1.6, and 2, respectively. As seen, in all panels of this figure, by increasing the nonlinear parameter $\gamma$, the reflection coefficient decreases, and the transmission coefficient rises. In the non-fractional Schrödinger equations (Panel C), the transmission (reflection) coefficient monotonically decreases (increases) by increasing the barrier width. However, by reducing sufficiently the fractional parameter $\alpha$, this behavior is no longer monotonic. In Panel A, the transmission (reflection) coefficient first decreases (increases) and then increases (decreases) by increasing the barrier width. Thus, the effect of the barrier width on the transmission and reflection coefficients is surprisingly non-monotonic. Another fact is that, for the non-fractional Schrödinger equation (Panel C), for the width more than a critical barrier width (width_critical_ ≈ 8), the transmission and reflection coefficients are constant extrema (for $\gamma$ = 0, R ≈ 1 and T ≈ 0), which it seems conspicuous, but this is not true for the fractional Schrödinger equation. This means that in the fractional Schrödinger equation for any widths, and $\gamma$, there are non-zero and non-unique values for the transmission and reflection coefficients, respectively.

**Figure 5:**
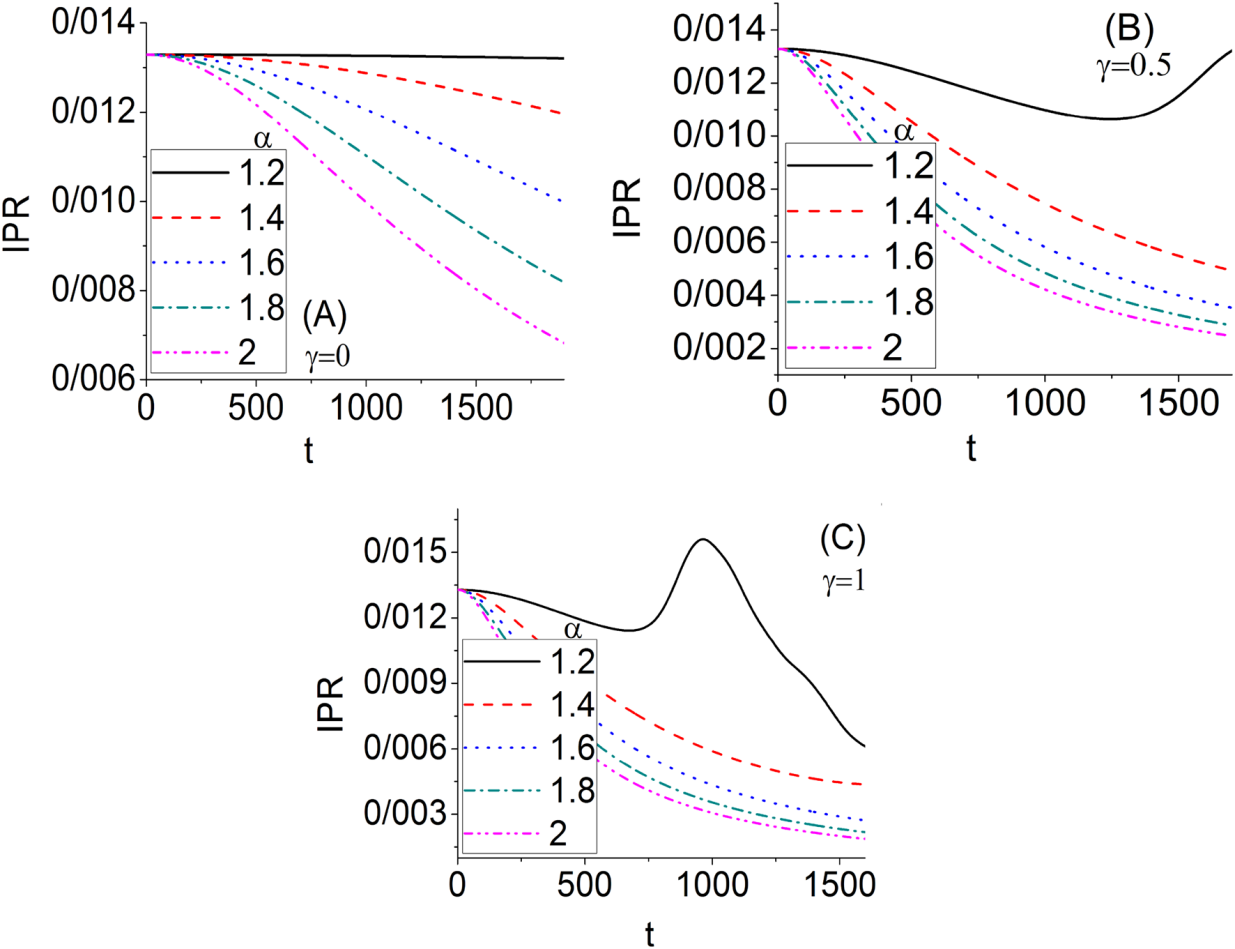
The variation of IPR as a function of time for $\kappa = 0.5$. The different values of the fractional order $\alpha$ and nonlinear parameter $\gamma$ have been examined.

Figure 5 shows the variation of IPR as a function of time. The different values of the fractional order $\alpha$ and nonlinear parameter $\gamma$ have been examined in three panels. Panel A is related to the linear fractional and non-fractional systems, while Panels B and C are associated with the nonlinear fractional and non-fractional systems. It is well known that the IPR of an extended state (metallic phase) goes to zero in the thermodynamic limit, but for a localized state (insulator phase), IPR is finite even in the thermodynamic limit^1^. Therefore, as this figure shows, the non-fractional systems have the metallic phase for both linear and nonlinear characteristics. However, the linear and intermediate nonlinear strongly fractional systems have the insulator phase. On the other hand, strongly nonlinear and non-fractional systems go to the metallic phase from the insulator phase by increasing time. The fractional characteristic is an essential factor because the non-fractional systems, for any value of $\gamma$, can only have the extended phase. This means that the wave packet survival (localized phase) can be observed for a long time only if we assume the strongly fractional systems. The wave packet survival that can lead to solitonic characteristics is an essential issue in communication technologies^42^.

**Figure 6:**
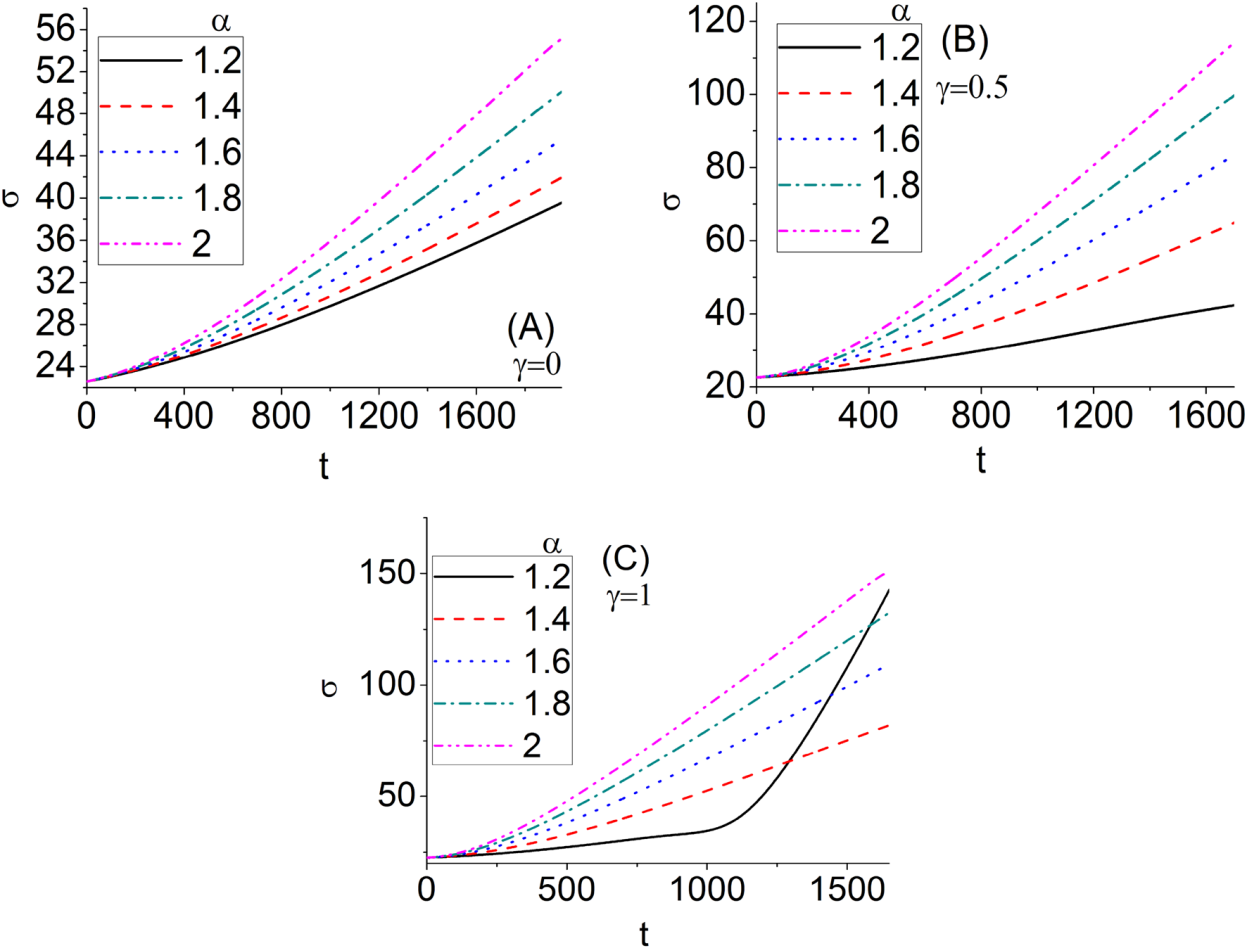
The variation of the second moment $\sigma$ as a function of time for $\kappa = 0.5$. The different values of the fractional order $\alpha$ and nonlinear parameter $\gamma$ have been investigated.

Figure 6 shows the variation of the second moment $\sigma$ as a function of time. The different values of the fractional order $\alpha$ and nonlinear parameter $\gamma$ have also been investigated. The mean-square displacement (second moment) increases by increasing time. For a fixed given $\gamma$, the second moment increases by increasing $\alpha$ except for $\gamma$ = 1 for the times more than about 1300, which that means $\sigma$ behaves differently for the strongly nonlinear systems at great times. Also, for a fixed given $\alpha$, the second moment increases by increasing $\gamma$. Thus, the wave packet spread faster in the less fractional and more nonlinear systems.

## Conclusion

In this study, we investigated the reflection, trapping, and transmission of a traveling Gaussian wave packet through a rectangular quantum barrier. We showed that the fractionality did not destroy the Gaussian shape of RP-IGW and TP-IGW, but the nonlinearity changed it. In the fractional Schrödinger equation, the RP-IGW and TP-IGW spread more slowly. The wave packet traveled more rapidly in the systems with smaller effective masses. By simultaneous use of the fractional and nonlinear terms in the Schrödinger equation, sharp shock pulses could be created. By using a small barrier height and small fractional parameter as well as a large nonlinear parameter, some parts of the incident Gaussian wave packet are trapped in the barrier region. Surprisingly, for any barrier height, the transition coefficient of the fractional Schrödinger equation was non-zero for nonlinear and enough fractional systems. Also, for any barrier width and nonlinear fractional systems, it was non-monotonic with respect to the barrier width, so it was not zero at all. The localized state could be achieved only for a strongly fractional system, which is so essential in soliton propagation (Supplementary Video 1). As a result, the outcomes of this paper can be a great help to improve wave propagation physics.

### Supplementary Information

Supplementary Video 1.

Supplementary Video 2.

Supplementary Video 3.

Supplementary Video 4.

Supplementary Video 5.

Supplementary Video 6.

Supplementary Video 7.

Supplementary Video 8.

Supplementary Video 9.

Supplementary Video 10.

Supplementary Video 11.

## Data Availability

The data that support the findings of this study are available from the corresponding author upon reasonable request.
